# Intention understanding over T: a neuroimaging study on shared representations and tennis return predictions

**DOI:** 10.3389/fnhum.2014.00781

**Published:** 2014-10-06

**Authors:** Stephanie Cacioppo, Frederic Fontang, Nisa Patel, Jean Decety, George Monteleone, John T. Cacioppo

**Affiliations:** ^1^Department of Psychiatry and Behavioral Neuroscience, The University of ChicagoChicago, IL, USA; ^2^High-Performance Electrical NeuroImaging Laboratory, Center for Cognitive and Social Neuroscience, The University of ChicagoChicago, IL, USA; ^3^Tennis AcademyPau, France; ^4^Department of Graduate Nursing, Western University of Health SciencesPomona, CA, USA; ^5^Department of Psychology, Brain Imaging Center, The University of ChicagoChicago, IL, USA

**Keywords:** embodied cognition, biosocial interaction, dyads, intention understanding, shared representation, fMRI, social neuroscience, mirror neuron system

## Abstract

Studying the way athletes predict actions of their peers during fast-ball sports, such as a tennis, has proved to be a valuable tool for increasing our knowledge of intention understanding. The working model in this area is that the anticipatory representations of others' behaviors require internal predictive models of actions formed from pre-established and shared representations between the observer and the actor. This model also predicts that observers would not be able to read accurately the intentions of a competitor if the competitor were to perform the action without prior knowledge of their intention until moments before the action. To test this hypothesis, we recorded brain activity from 25 male tennis players while they performed a novel behavioral tennis intention inference task, which included two conditions: (i) one condition in which they viewed video clips of a tennis athlete who knew in advance where he was about to act/serve (initially intended serves) and (ii) one condition in which they viewed video clips of that same athlete when he did not know where he was to act/serve until the target was specified after he had tossed the ball into the air to complete his serve (non-initially intended serves). Our results demonstrated that (i) tennis expertise is related to the accuracy in predicting where another server intends to serve when that server knows where he intends to serve *before* (but not *after)* he tosses the ball in the air; and (ii) accurate predictions are characterized by the recruitment of both cortical areas within the human mirror neuron system (that is known to be involved in higher-order (top-down) processes of embodied cognition and shared representation) and subcortical areas within brain regions involved in procedural memory (caudate nucleus). Interestingly, inaccurate predictions instead recruit areas known to be involved in low-level (bottom-up) computational processes associated with the sense of agency and self-other distinction.

## Introduction

“If there is something you don't want to be on a tennis court, it is predictable,”

John McEnroe, ESPN, U.S Open, 8-30-13.

The way in which athletes read and anticipate the actions of their opponent during fast-ball sports, such as a tennis, is a challenging and complex process that is a remarkable feat in itself. A tennis player's ability to predict an opponent's intentions quickly and accurately is particularly important during the return of serves, where the time required to plan and initiate a response typically exceeds the flight time for the ball (Glencross and Cibich, 1977; Farrow and Abernethy, 2003; Williams et al., 2004; Wright and Jackson, 2007). Given the high speed a ball can reach nowadays (e.g., above 130 mph), the receiver must make a decision regarding the direction of his/her opponent's serve intentions (e.g., to serve to the center of the tennis court or T, the middle of the service box, or the wide side of the service box) based, at least in part, on information identified prior to the server striking the ball (Wright and Jackson, 2007).

Several studies have investigated the mechanisms underlying predictive motor skills in such time-constrained situations (Williams et al., 2004). For instance, it has been shown that expert tennis players, when compared to their less skilled counterparts, are: (i) better at detecting advance (i.e., early or pre-event) information from an opponent's postural orientation (Williams et al., 2002); (ii) have more efficient visual search behaviors (Goulet et al., 1988, 1992; Singer et al., 1996; Helsen and Starkes, 1999; Overney et al., 2008); (iii) pay more attention to motion information (Williams et al., 2002); and (iv) possess greater knowledge/expertise of situational probabilities (Williams et al., 2004). Although the past decade has been characterized by a growing body of research dedicated to better understand the factors playing a role in anticipation and predictive skills in fast-ball sports, very few studies have examined their underlying neural mechanisms (e.g., Wright and Jackson, 2007). Among these studies, Wright and Jackson (2007) used temporal occlusion to study the neural bases of action prediction in fast-ball sports. Relative to a passive condition, action prediction recruited notably a fronto-parietal network (Wright and Jackson, 2007), which is known to involve the putative human mirror neuron system (hMNS; Grafton et al., 1996; Rizzolatti and Sinigaglia, 2008; Rizzolatti and Fogassi, 2014). They extended this result by demonstrating that experts, compared to novices, tend to show stronger brain activation within the hMNS for early-occluded than for late-occluded time sequences of a tennis shot (Wright and Jackson, 2007).

Theories of simulation and embodied cognition provide a neural basis for such early predictive ability in experts by specifying the involvement (and re-activation) of the inferior fronto-parietal network (possibly including the hMNS), which is known to be activated by one's own motor performance as well as perspective taking, sensorimotor integration, and procedural memory (Rizzolatti et al., 1996; Blakemore and Decety, 2001; Ruby and Decety, 2001; Ruby et al., 2002; Buccino et al., 2004; Rizzolatti and Sinigaglia, 2007, 2008; Grafton, 2009; Grafton et al., 2009; Ortigue et al., 2009a,b, 2010a; Juan et al., 2013; Tomeo et al., 2013; Rizzolatti and Fogassi, 2014). Such research has highlighted two overlapping neural networks, known as the action observation network (AON) and the social network (SN), that are differentially involved in the process of understanding intentions and actions (Grafton, 2009; Ortigue et al., 2009a,b, 2010a; Juan et al., 2013). The AON, which contains a subset of regions within the hMNS including the posterior superior temporal sulcus (STS), the inferior parietal lobule, and the inferior frontal gyrus, has been linked to perception of actions and understanding intentions utilizing embodied cognition (Grafton et al., 1996; Desmurget and Grafton, 2000; Rizzolatti and Craighero, 2004; Rizzolatti and Sinigaglia, 2007, 2008; Desmurget et al., 2009). On the other hand, the SN includes the medial prefrontal cortex, STS, precuneus, insula, and amygdala, and has been linked to perceiving biological motion and theory of mind attribution (Frith and Frith, 1999; Allison et al., 2000; Wheatley et al., 2007; Decety and Cacioppo, 2012). Theories of embodied cognition and simulation suggest that the emulation of these two brain networks contributes to the capacity to read and predict the intentions of others.

Although embodied cognition is not a prerequisite to act or to understand others' actions, simulation theories suggest that the more these observed actions are congruent with integrated templates of past self-related motor experiences, the easier it is to read these observed actions and intentions as the actor and the observer share a mental map of the action (Niedenthal et al., 2005; Niedenthal, 2007). In line with this, the model of shared representation suggests that sport mates or close partners develop a “transactive” mental representation of their self while acing—a mental representation that calls for cognitive interdependence and includes a structure of stored information across the two individuals (Wegner et al., 1985; Ortigue et al., 2010b). Cognitive interdependence in dyads relates to the concept of inclusion of the other in the self-mental representation—a concept that is closely tied to self-expansion mechanisms, and embodied cognition. Although we are all interdependent to some degree, the model of shared representation highlights the extent to which partners may implicitly read and influence each other's perceptions of their actions, emotions, and intentions. Cognitive interdependence can provide a processing advantage during anticipatory representations of others' behaviors, such that sport mates (e.g., competitors) are more efficient and more frequently successful in forming shared mental representations based on internal predictive models of actions (Ruscher et al., 2003).

In the context of sport athletes, intention understanding among peers is based, in part, upon a shared mental representation of actions, with sport mates being able to better anticipate one another's actions due to greater experience observing each other's actions in different situations or due to shared experience in a specific sport (Wegner et al., 1985; Hommel et al., 2001; Ruscher et al., 2003; Agnew and Etcheverry, 2006). As an illustrative case in point, previous studies have demonstrated that experts in a sport (e.g., basketball, dance, or soccer) are better and/or faster (than novices) at understanding intentions of an opponent or a teammate just by watching their body movements (Jeannerod, 2001; Wolpert et al., 2003; Calvo-Merino et al., 2005; Cross et al., 2006, 2009; Aglioti et al., 2008; Abreu et al., 2012; Tomeo et al., 2013). Moreover, evidence suggests that intention perception may be facilitated not only by congruence between observed and past actions (Niedenthal et al., 2005; Niedenthal, 2007; Aglioti et al., 2008; Ortigue et al., 2010a,b; Tomeo et al., 2013), but also by the emotional bond between actor and perceiver, with a stronger bond associated with better (Cutting and Kozlowski, 1977) or faster intention understanding (Ortigue et al., 2010b). This facilitation effect putatively occurs through a direct, fast and automatic visuo-motor matching process between what the experts see and what they have executed (over and over) in the past.

Reciprocally, this model of shared representation predicts that this facilitation effect fades away when an actor plans their actions with an unusual mental representation of their intentions (as it can be the case in fool actions). In a recent behavioral study, Tomeo and colleagues tested this notion by manipulating the congruence between a soccer kicker's bodily movements and the subsequent ball trajectory and investigated the prediction performance from 16 kickers, 16 goalkeepers, and 16 novices (Experiment 1; Tomeo et al., 2013). Their results showed that kickers were more often fooled than goalkeepers and novices during incongruent actions, although both types of experts (kickers and goalkeepers) outperformed novices (Tomeo et al., 2013). This study reinforced the model of shared representation by demonstrating that: (i) shared expertise plays a crucial role in intention anticipation (which has also been demonstrated previously by a large body of work), and that (ii) previously shared mental representation among peers (kickers-to-kickers) may hinder intention anticipation when one of them performs deceptive actions. However, because an actor always knows in advance what their intentions are (whether they are deceptive intentions or not) and so may display visual cues that are shared with their observers, one may argue that this study does not fully test the model of shared intentions.

To address this argument, we designed a novel behavioral intention inference task (IIT), which included two conditions: (i) one condition showing video clips of an unfamiliar right-handed tennis athlete (hereafter the server) who knew in advance where he was about to act/serve (*initially intended action*, IIS condition; see Supplementary Movie 1 for a sample) and (ii) another condition showing video clips of that same athlete when he did not know in advance where he was about to act/serve (*non-initially intended serve*, NIIS condition; see Supplementary Movie 2 for a sample). In this NIIS condition, an experimenter (NP) told the server where to serve (either to the center of the tennis court or to the wide side of the service box) *after* he had tossed the ball into the air to serve. Although the voice of the experimenter, NP, was recorded in each video for quality and accuracy control purposes, participants watched silent video clips. In all video clips the server bounced the ball twice, tossed the ball, and finally stroke it to perform a series of serves.

**Figure 1:**
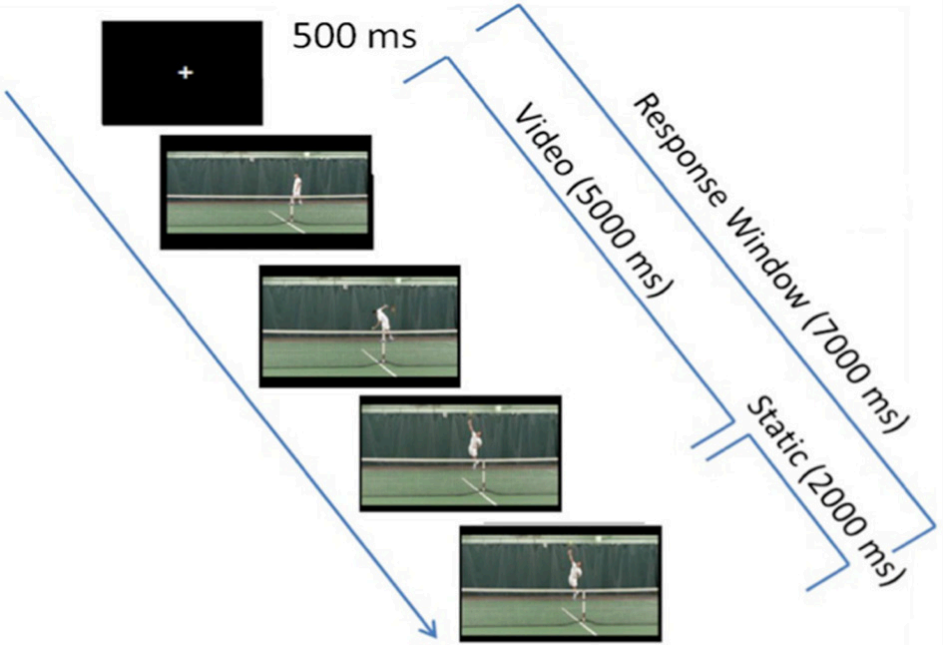
**Experimental paradigm**. Each trial first consisted of a 500 ms-fixation cross, followed by a 5-s video clip, which froze on the last frame of the video clip (when the player's racquet struck the ball) for an additional 2 s in order to provide participants with more response time, if needed. In each video clip, the tennis player was shown bouncing the ball twice, then winding up and striking the ball.

**Figure 2:**
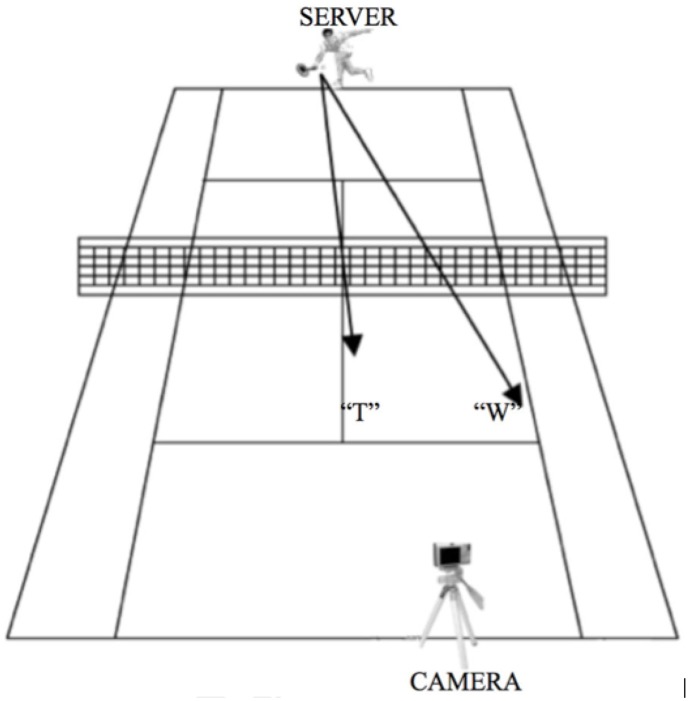
**Experimental layout during creation of the stimuli**. The camera was located on a tripod on the baseline next to the service box of the court diagonal from where the tennis player stood. The tennis player could serve either to the center of the opposite side of the tennis court (“T”) or to the wide side of the service box (“W”). This setting allowed the participants to have a first person view while watching the video clips, as if they were in a match situation standing on the tennis court ready to receive the serve.

We recorded brain activity from 25 male tennis experts (hereafter, the observers) while they were performing this tennis IIT (*t*IIT; Figure 1) and were asked to predict the ultimate direction (either to the T or to the wide side of their service box; see Figure 2 for an illustration of these two serve directions). As in previous studies (Williams et al., 2004), serves that landed in the middle of the service box were not considered, because of the potential difficulty to classify objectively an observer's response to such serves. No feedback was provided to the participants during the *t*IIT. The participants were not aware that the server was performing this series of serves under the above two different conditions (*initially intended serves and non-initially intended serve*). Because, the ability to evaluate the body movements of another player and predict their intentions is facilitated by coupling previous experiences (both the sensorimotor cues that diagnose serve location and the sensorimotor cues that do not) with any sensory input (e.g., visual cues) that might cue them as to the intended action (Kording and Wolpert, 2004; Kilner and Frith, 2007; Kilner et al., 2007), we assumed that, in the absence of reliable sensory information due, for instance, to the high speed of a tennis serve, expert tennis players would rely more on their previous bodily visuo-motor experiences to determine the intended location of the serve than visual information (Kording and Wolpert, 2004). It also follows that when an opponent completes most of the action (e.g., a tennis serve) without any clear intention in mind, expert tennis players should be near or at chance level when trying to predict the direction of that action.

Interestingly, in the case of a tennis match, the same action (e.g., a tennis serve) may reflect different intentions (e.g., to serve to the T or the wide side of the service box). Given the importance of not being predictable on the tennis court, expert tennis players are taught to perform the same perceptible actions regardless of their service intentions. Therefore, expert tennis players not only have to rely on their past experiences of serving in tennis to predict the intended location of their opponent's serve but they must eschew any masking behavioral cues that would hide the intention of their opponent. The study of expert tennis players while they try to predict accurately the ultimate intentions (direction of a serve) of another player, thus, constitutes a unique and ecologically valid opportunity to better understand the mechanisms underlying the assumed visuo-motor matching in embodied cognition.

## Materials and methods

### Participants

All 25 fMRI participants were right-handed (Edinburgh Handedness Inventory, Oldfield, 1971), and had normal or corrected to-normal visual acuity with no history of psychiatric or neurological disorder (as ascertained by a brief anamnesis and self-report questionnaires, Davis, 1980; Zigmond and Snaith, 1983; Spielberger, 1987; Russell, 1996; Bellini et al., 2002; Lamm et al., 2007). Mean age of participants was 26.2 years (*SD* = 8.95). Because the goal of this study was to test the theory of shared representation among experts, all participants were expert tennis players (see questionnaire section below for further details).

### Procedure

Prior to participation, volunteers provided written informed consent that had been approved by the Ethical Committee of the University of Chicago, Illinois. The study took place over a single visit. Upon arriving at the University of Chicago Brain Imaging Center, participants completed a series of standard screening forms. Then, they completed a series of tennis-related questionnaires, and a brief practice of the tennis intention inference task (practice *t*IIT) outside the scanner, and an experimental tennis intention inference task (experimental *t*IIT) while they were in the 3T Philips fMRI scanner (see below for further details).

### Questionnaires

Participants' tennis expertise was ascertained by their United States Tennis Association (USTA) playing level, which was, on average, 4.24 (*SD* = 0.93) out of a 7-point scale. A USTA level of 4 denotes a player who has a dependable forehand and backhand, a variety of good shots (such as serves and lobs), and good team work in doubles. To further understand the participants' tennis profile, a series of exploratory questions about their tennis habits/behaviors were also asked to the participants. These additional questions investigated: (a) the number of hours our participants played tennis per week; (b) the number of hours they watched tennis on TV per week; (c) the age when the participants first begun playing tennis; and (d) the number of hours they played tennis on video games per month.

### Practice task tennis intention inference task (practice tennis IIT)

In the practice *t*IIT, participants received detailed instructions regarding the task, and viewed one sample video clip for which they were asked to determine the intended location (either to the center of the tennis court i.e., to the ≪ T ≫, or to the wide side of the service box i.e., to the ≪ W ≫) of the serve depicted in that video clip. This example was meant to allow participants to understand fully the instruction they would then perform in the experimental tennis IIT (*t*IIT).

### Experimental tennis intention inference task (experimental *t*IIT)

As in the practice *t*IIT, in the experimental *t*IIT participants were instructed to indicate as rapidly and as accurately as possible where the tennis server intended to serve (either to the “T” or to the “W” side of the service box).

Each trial began with a 500 ms-fixation cross that was followed immediately by a 5 s-target video clip (Figure 1). Because previous research has shown that expert athletes respond to visual cues that occur well before a shot is struck (Ward et al., 2002), all video clips in the *t*IIT (including video clip used in the practice *t*IIT) ceased as the expert tennis player's racquet contacted the ball, and remained frozen on this image for a maximum of 2 s i.e., until the end of the duration of the response window, which started when the video started. As during the brief practice and as in previous IIT studies (Ortigue et al., 2009a,b, 2010a,b), participants were allowed to respond at any point during the video clip presentation, as well as during the 2 s immediately following the completion of the video clip for a total response window of 7 s (Figure 1).

### Stimuli

Stimuli consisted of eight video clips [2 types of serves (IIS and NIIS) × 2 starting positions (left and right) × 2 ball landing sides (to the center “T” and to wide side “W” of the service box)] of an unfamiliar right-handed male expert tennis player (from Syracuse University, Upstate New York) performing a tennis serve (one per video). The video clips showed the tennis player on two different starting positions [standing either on the right side from the participant's perspective of the tennis court (half of the video clips), or on the left side of the tennis court] in order to control for any participants' lateralized attentional bias during the experimental *t*IIT.

Across serve conditions, the server was able to perform the same movements repeatedly, independently of the ultimate outcome of his serves (either to the center or the wide side of the service box) because of his high tennis level (USTA level: 7, which denotes a world class player). He was also able to bounce the ball, toss it and serve in the same way, using his regular action with a relatively consistent velocity, independently of the intentionality manipulation (see Supplementary Movies 1, 2 for examples).

### Apparatus

Videos of the server were taken with a digital Sony Cybershot camera. The camera was located on a tripod on the baseline next to the service box of the court diagonal from where the tennis player stood (Figure 2). This setting allowed the participants to have a first person view while watching the video clips, as if they were in a match situation standing on the tennis court ready to receive the serve. All video clips were presented using E-Prime 2.0 (Psychology Software Tools Inc., Pittsburgh, USA). During the scanning session, participants viewed the stimuli on a back projection screen mounted on the head coil of the MRI scanner.

### Validation of the stimuli and viability of the paradigm

In order to test the viability of our novel paradigm and test the similarity of the server's movements across serve conditions, we performed three different steps. First, we performed a quantitative analysis of all the tennis video clips using Dartfish i.e., a performance video analysis software. Extensive research on anticipatory skills in sport, in which the visual information available to understand a tennis serve is cut off at some specific time frames (temporal occlusion) during the serve, indicates that a key event for tennis is the ball/racket contact, with the movement of the arm and the racket prior that key event being the source of critical cues for racket sports (Tenenbaum et al., 2000; Shim et al., 2006; Abernethy and Zawi, 2007; Jackson and Mogan, 2007; Wright and Jackson, 2007; Williams et al., 2009 for review). Therefore, we analyzed the average speed of the server's movement up until he hit the ball. Results revealed that this average speed did not significantly vary between the IIS and NIIS conditions [*M_IIS_* = 1.94 m/0.2 s, *SD* = 0.13; *M_NIIS_* = 2.03 m/0.2 s, *SD* = 0.04; *t*_(6)_ = −1.44; *p* =.20; two-tailed]. This quantitative analysis revealed that the stimuli from both conditions were visually comparable in terms of speed of the server's movements.

Second, we performed a visual qualitative analysis of the video clips by asking three persons (SC, JTC, BM) who are knowledgeable (although non expert) in tennis to view all the video clips, one by one, and tried to determine whether any obvious visual differences appeared between the two serve conditions i.e., IIS and NIIS. Although these three persons were aware of the two different experimental conditions, none of them was able to identify any visual differences between video clips. This result was reinforced with the behavioral performance from 29 other individuals [18 men, 11 women; mean age of 31.55 (*SD* = 10.32)] we recruited on Amazon Mechanical Turk (MTurk). To ensure the respondents were real participants, rather than a computerized script, a compliance check question was included that instructed respondents to answer “left” to validate that they were reading the survey prior to responding. Although 50 individuals were initially involved, 42% of the respondents failed to answer this compliance check correctly, so their data were not included in the analysis. Thus, the final sample was composed of 29 individuals.

All 29 participants were non-expert tennis players (as ascertained by their self-report USTA tennis levels: *M* = 2.73, *SD* = 1.29) and were not aware of the two serve conditions. Each participant viewed all video clips in one of two specific randomized orders. Results revealed no significant gender difference [for men: *M_IIS_* = 51.4%, *SD* = 18.1; *M_NIIS_* = 52.8%, *SD* = 28.3; for women: *M_IIS_* = 56.8%, *SD* = 22.6; *M*_*NIIS* serves_ = 52.3%, *SD* = 28.4; *t*_(27)_ = −0.367, *n.s., r* = −0.073], and no significant effect of the serve conditions on accuracy [*t*_(28)_ = 0.138, *n.s., r* = 0.018], with participants being at chance level for both types of serves (*M_IIS_* = 53.5%, *SD* = 19.7; *M_NIIS_* = 52.6%, *SD* = 27.8). No reaction times were recorded in the MTURK study.

Then, to make sure that this novel task was suitable for tennis experts, we asked a pro-tennis player (FF), who is also an active pro-tennis coach on the ATP tour, to watch the video clips and perform a qualitative analysis. Although he was not aware of the two conditions, he was able to detect nuances at the level of the hips of the server that differed between the two conditions. Interestingly, he was not able to name or identify the two conditions after identifying two different types of stimuli. All he could report was that some tennis serves (the IIS, according to SC's observation of FF's performance) were easier to anticipate than others in the set of video clips. This procedure suggests that a pro-tennis player could *not* report the specific content (IIS and NIIS) of the video clips, and that we, thus, could use these video clips in our study with tennis experts.

### Experimental paradigm

The present *t*IIT included 5 blocks. Each block was composed of 8 trials. In order to increase the number of stimulus presentations, the participants were asked to perform the experimental *t*IIT twice: once in the hypothetical context of a friendly match with a tennis practice partner; and once in the hypothetical context of a competitive match with that same tennis practice partner. This procedure led to a total of 10 blocks. Block order was selected at random by E-Prime 2.0 (Psychology Software Tools Inc., Pittsburgh, USA), with the condition that both match types must be presented exactly five times. Accordingly the order of presentation of the 10 blocks differed across participants. All the eight videos were presented once in each of the 10 blocks. The presentation of each video was completely randomized within each of block. In sum, a total 80 trials were presented to each participant.

### Dependent measures

Response accuracy (in percent, %), reaction times from the onset of the video (in milliseconds, ms), and brain activity were recorded while participants made a decision as to ultimate direction of the serves. In addition, to account for a potential intention advantage, we calculated a conventional accuracy index score (Marshall et al., 1975) for percentage correct responses between Initially Intended Serve (IIS) with Non-Initially Intended Serve (NIIS) condition as follows: (IIS − NIIS)/(IIS + NIIS). Thus, positive values indicate an IIS advantage and negative values a NIIS advantage. Finally, to eliminate any potential response bias in our accuracy measure, we also calculated a *d*' accuracy index (Dprime.AccuracyIndex), using Marshall et al.'s accuracy Index formula as follows: (IIS − NIIS)/(IIS + NIIS). In this *d*' accuracy index, we converted percent correct scores to *d*' for IIS and NIIS items using the formula z(Hit) − z(False Alarm), which gave us Dprime.IIS and Dprime.NIIS. Converted Dprime values.

### Behavioral statistical analyses

In line with our hypotheses, we collapsed across match hypothetical contexts (friendly or competitive), starting position (left or right) and ball landing sides (“T” or “W”), yielding a repeated-measures design with serve type (IIS vs. NIIS) as a within-subjects factor. Mean reaction times and percentage were calculated for each subject and condition. Outliers were removed by eliminating responses greater than 3.5 standard deviations from the grand mean. Using this cutoff resulted in the removal of 4.5% of all trials (across participants). Repeated measures ANOVAs were utilized to analyze potential differences in reaction times and accuracy between serve types in the *t*IIT. Additionally, correlational analyses were performed to examine the relationship between self-reported data about tennis (USTA level, hours playing tennis per week, hours watching tennis on TV per week, and age first learned tennis) and our behavioral dependent measures (See Table 1 for accuracy and reaction times; and see Table S1 for accuracy index and *d*' accuracy index).

**Table 1:** **Pearson correlations (*df* = 23) between various tennis-related self-reported measures and behavioral measures**.

	**RT_all_**	**RT_IIS_**	**RT_NIIS_**	**ACC_all_**	**ACC_IIS_**	**ACC_NIIS_**
USTA level	0.24	0.30	0.22	−0.09	0.12	−0.23
Hours playing tennis/week	−0.15	−0.13	−0.18	0.44^*^	0.43^*^	0.24
Hours watching tennis/week	0.17	0.19	0.15	0.17	−0.05	0.05
Age first learned tennis	−0.03	−0.06	−0.004	−0.31	−0.26	−0.23

* *Denotes p < 0.05*.

### Neuroimaging recordings and processing

#### Magnetic resonance imaging recordings

Imaging was performed on a 3-T Philips Achieva Quasar Dual 16 Ch scanner with quadrature head coil used for spin excitation and signal reception. High-resolution volumetric T1-weighted spoiled gradient-recalled (SPGR) images were obtained for each participant in one hundred sixty-one 1.0-mm sagittal slices with 8° flip angle and 24 cm field of view (FOV) for use as anatomical images. Functional images using a block design and were acquired using a echo-planar acquisition with Z-Shimming with 32 × 4-mm coronal slices with an inter-slice gap of 0.5 mm spanning the whole brain (*TR* = 2.5 s, *TE* = 30 ms, flip angle = 80°, FOV = 22 cm, 64 × 64 matrix size, fat suppressed).

#### Functional image processing and analyses

Image pre-processing and analyses were performed using Analysis of Functional NeuroImages software (AFNI, Medical College of Wisconsin). For each participant, motion detection and correction were undertaken using a six-parameter, rigid-body transformation. Functional images were co-registered and spatially smoothed using a 5-mm full width at half maximum Gaussian filter. Individual-subject analyses were conducted using the general linear model to generate estimates of blood oxygenation level-dependent (BOLD) signal on a voxelwise basis (Ward, 2002). Stimulus timing vectors for each of the experimental conditions were modeled for 3 TRs after each stimulus onset, and each vector was convolved with a gamma-variate waveform using the AFNI program Waver. The resulting model was fit voxelwise to preprocessed time-series data with a linear least-squares model using the AFNI program 3dDeconvolve, generating a map consisting of beta coefficients (fit values) at each voxel for each modeled condition—intended serve/non intended serve;—as well as a baseline coefficient. Two GLMs were assessed for each subjects: one modeling only correctly identified T/W trials, and one modeling only incorrectly identified T/W trials. Output from the deconvolution analysis for each subject was scaled voxelwise to percent signal change from baseline, and each subject's data were spatially transformed to the MNI Colin27 Atlas (1998) stereotaxic coordinate space and interpolated to 3 mm^3^ isometric voxels for group analysis (Holmes et al., 1998). Voxelwise fMRI analyses were performed at the group level, the results of which were corrected for multiple comparisons by using a Monte Carlo simulation to determine minimum cluster sizes corresponding to an alpha value of 0.05 for voxelwise thresholds of *p* <.01 and *p* <.025 (Nichols, 2012). Groupwise analyses were carried out for correct-item and incorrect-item models. A voxelwise [IIS/NIIS] ANOVA was performed, as well as whole-brain voxelwise Pearson correlation to identify regions in which differential BOLD activity in response to the stimulus effect (i.e., IIS-NIIS) was associated with behavioral responses related to tennis i.e., USTA level, hours playing tennis per week, hours watching tennis on TV per week, and age first learned tennis. In addition, we used the reaction times and intention accuracy index scores (**Tables 4**, **5**) in correlational analyses with the BOLD signal contrasting IIS and NIIS (Voxelwise correlation of BOLD [IIS - NIIS] for correct trials × Dprime.AccuracyIndex, cluster were also calculate with a threshold at voxelwise *p* <.01 corrected to alpha < 0.05. See Table S2).

## Results

### Questionnaires

On average, participants reported: (a) having a USTA level of 4.24 (*SD* = 0.93) out of a 7-point scale; (b) playing tennis 3.56 (*SD* = 3.98) hours per week, (c) watching tennis on TV 1.92 (*SD* = 4.31) hours per week, and (d) having begun playing tennis at the age of 9.36 (*SD* = 5.26). Most of the participants also reported not playing tennis on video games (three of the participants reported playing video-tennis game less than 2 hours per month).

#### Behavioral tIIT results

As predicted, the behavioral results showed that the observers were better at predicting initially intended serves, IIS (64.16% correct, *SD* = 0.099) than non-initially intended serves, NIIS [52.40% correct, *SD* = 0.099; *F*_(1, 24)_ = 25.387; *p* <.001; *d* = 1.18]. When percent correct values were converted to *d*', Z(H) − Z(FA), results showed a similar significant effect of IIS/NIIS on accuracy [*t*_(24)_ = 4.736, *p* = 8.126e-05; 95% CI (0.34; 0.87); mean of *d*' differences = 0.61]. Note that this behavioral result is also statistically significant on the same order of magnitude (*p* <.0001), when using percent correct in lieu of *d*'. No differences in reaction times were observed between the two serve conditions for correct responses [*M_IIS_* = 5609.64 ms, *SD* = 387.58; *M_NIIS_* = 5637.26 ms, *SD* = 425.12; *F*_(1, 24)_ = 2.62; *p* =.12; *d* = −0.07). A *post-hoc* paired *t*-test conducted to see if reaction times were different between IIS and NIIS for incorrect responses revealed no differences in reaction times between the two serve conditions [*M*_*IIS*. incorrect_ = 5622.75 ms, *SD* = 418.99; *M*_*NIIS*. incorrect_ = 5636.25 ms, *SD* = 438.77; *t*_(24)_ = −0.61; *p* =.55; *d* = −0.031]. To check whether the participants were able to identify the different conditions during the task, an experimenter (AB) performed a debriefing with each participant after the experimental *t*IIT. None of the participants reported being aware of the two serve conditions.

#### Behavioral correlational analyses

Significant positive correlations were observed between the number of hours the participants reported playing tennis per week and three behavioral measures: (i) the overall accuracy; (ii) the accuracy for IIS; and the *d*' accuracy index. Together these results suggest that the more participants reported playing tennis per week, the better they were at predicting accurately the tennis serve of the model, especially when this latter knew in advance where he intended to serve. No other significant correlations were found between the different tennis-related questionnaires and the accuracy or reaction times (See Table 1 and Table S1 for details).

### Neuroimaging results

#### IIS-NIIS contrast

**Figure 3:**
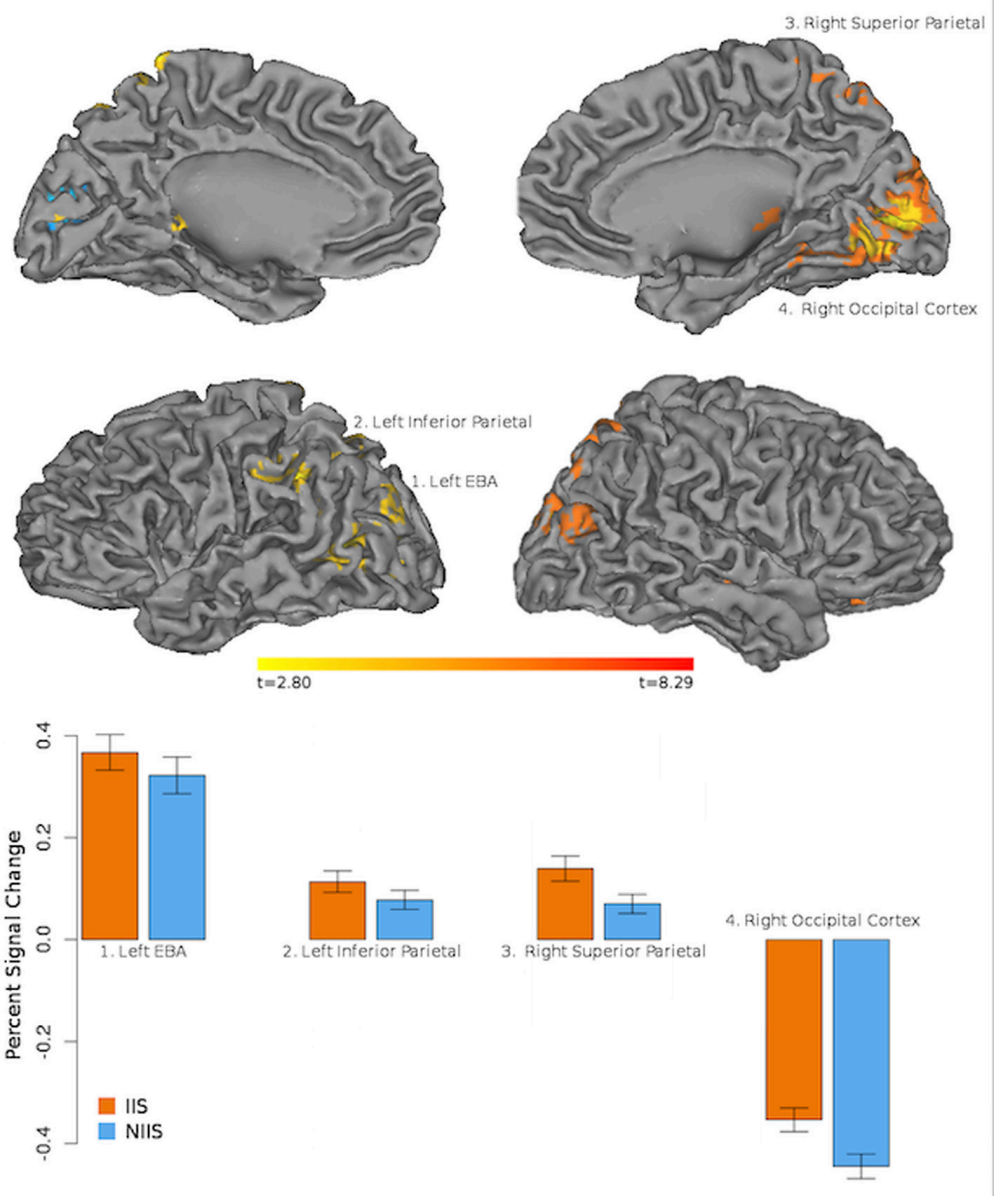
**Neuroimaging results representing the comparison between the IIS and NIIS contrasts for correct trials**. Results are projected onto the Caret AFNI Colin Brain surface model. Voxelwise threshold at *p* <.01, multiple comparison correction to minimum cluster vol of 702 ul (26 voxels). Results from the IIS - NIIS contrast are represented in orange/red. Results from the NIIS - IIS contrast are represented in blue.

In line with our behavioral results, our neuroimaging results showed regional changes in hemodynamic activity for correct behavioral predictions of initially intended (IIS) serves (compared to non-initially intended, NIIS, serves) in four main cortical areas: right occipital cortex, right superior parietal lobule (SPL), left extrastriate body area (EBA), and left inferior parietal lobule (IPL, extending to the left temporo-parietal junction, TPJ; Figure 3). In addition, increased brain activity was also detected in dopaminergic-rich sub-cortical regions (e.g., bilateral thalamus, right putamen, and right caudate nucleus; Table 2) known to be involved in somatosensory integration, motivation, goal-directed actions as well as formation habits and procedural memory (Ashby and Crossley, 2010, 2012). On the other hand, the brain activity associated with correct behavioral predictions of non-initially intended serves (compared to initially intended serves) revealed only one specific hemodynamic increase in the left calcarine gyrus, extending to the left cuneus—a brain region associated with visual information processing.

**Table 2:** **BOLD responses obtained for IIS > NIIS for correct trials**.

	**Vol (ul)**	***x***	***y***	***z***	***t***
27.8% overlap with Right Calcarine Gyrus (BA18)	15,579	18.2	−76.6	4.1	3.9245
24.5% overlap with Right Lingual Gyrus					
12.9% overlap with Right Cuneus					
11.1% overlap with Right Superior Occipital Gyrus					
10.9% overlap with Right Middle Occipital Gyrus					
5.3% overlap with Right Fusiform Gyrus					
32.2% overlap with Right Superior Parietal Lobule (BA7)	5832	2.8	−66.8	54.3	3.1006
23.9% overlap with Precuneus					
14.9% overlap with Superior Parietal Lobule					
12.1% overlap with Precuneus					
31.7% overlap with Right Thalamus	3105	27.7	−21.5	3	3.2895
11.7% overlap with Right Superior Temporal Gyrus					
7.7% overlap with Right Insula Lobe					
6.2% overlap with Right Putamen					
56.5% overlap with Left Thalamus	2646	−16.5	−23.5	7.9	3.325
10.9% overlap with Left Hippocampus					
78.1% overlap with Left Inferior Temporal Gyrus (BA37)	2538	−45.1	−63.9	2.4	3.2919
18.2% overlap with Left Middle Occipital Gyrus					
66.5% overlap with Left Inferior Parietal Lobule (BA40)	1512	−50.1	−42.7	35	3.2227
31.4% overlap with Left SupraMarginal Gyrus					
58.8% overlap with Right Putamen	1107	23.2	12.9	9.5	3.1579
23.5% overlap with Right Caudate Nucleus					
81.4% overlap with Left Middle Occipital Gyrus (BA18)	999	−31.7	−82.3	−4.5	3.1296
18.6% overlap with Left Inferior Occipital Gyrus					
94.5% overlap with Left Middle Occipital Gyrus (BA19)	891	−34.5	−83.1	20.9	3.1188
56.4% overlap with Right Inferior Frontal Gyrus (BA47)	756	25.8	32.3	−6.3	3.1743

*The overlap is the percentage of voxels relative to the entire size of the cluster that overlapped with a given region's spatial map in the AFNI MNI atlas. So, for example, “32.2% overlap in Right Superior Parietal Lobule” means 32.2% of the cluster's entire volume was spatially located in the MNI region outlining the Right Superior Parietal Lobule*.

**Figure 4:**
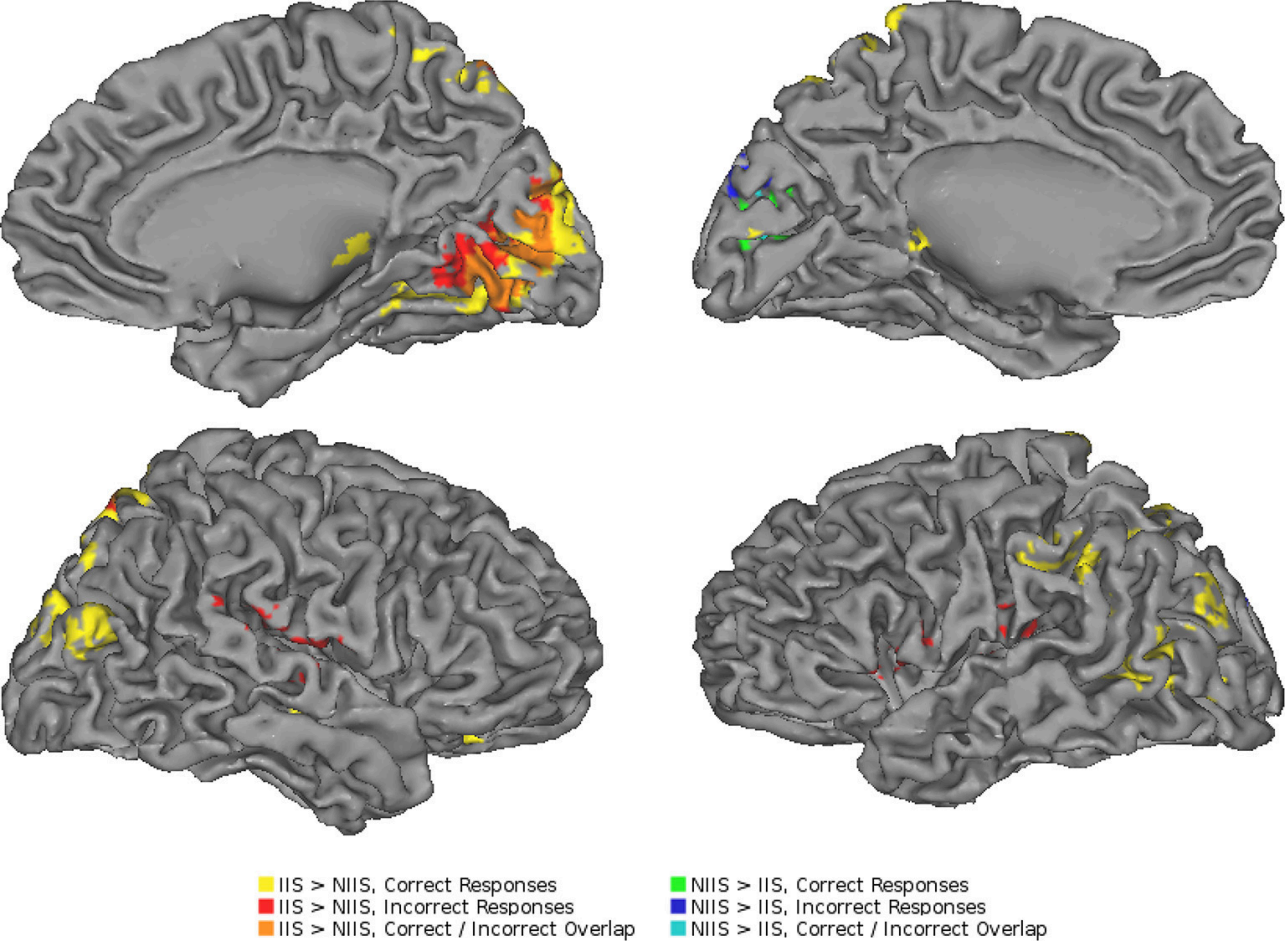
**Neuroimaging results representing the comparison between the IIS and NIIS contrast effects**. A surface projection on the Caret AFNI Colin Brain surface model depicting the two-tailed result of the main effects for IIS - NIIS for both correct and incorrect trials (*p* < 0.01, corrected). Color codes indicate cluster effects for correctly identified trials (yellow: IIS > NIIS, green: NIIS > IIS) and incorrectly identified trials (red: IIS > NIIS, blue: NIIS > IIS). Overlaps between these results are indicated with a combination of colors: orange regions indicate where correct and incorrect responses overlapped for IIS > NIIS contrasts in the right cuneus, while cyan indicates overlap of correct and incorrect NIIS > IIS effects in the left cuneus. No regions showed overlap between different directions of effects (i.e., IIS > NIIS and NIIS > IIS or vice-versa).

To further determine whether the above brain areas were specific to correct trials of the *t*IIT, we also performed an analysis of the brain activity of the participants' incorrect trials. As for the analyses of correct trials, the neuroimaging analyses of the regional changes in brain activity for *incorrect* behavioral predictions of IIS (compared to NIIS) revealed hemodynamic activation in brain areas involved in basic analysis of visual information and biological motion (right occipital cortex), attention (right SPL), and simulation and action observation (e.g., left inferior frontal gyrus, inferior parietal lobule; Table 3; Figure 4).

**Table 3:** **BOLD responses obtained for IIS > NIIS for incorrect trials**.

	**Vol (ul)**	***x***	***y***	***z***	***t***
40.9% overlap with Right Lingual Gyrus (BA18)	10,692	12	−72	0	4.26
40.9% overlap with Right Calcarine Gyrus					
9.1% overlap with Right Cuneus					
28.1% overlap with Left Rolandic Operculum (BA41)	1647	−38	−31	17	3.22
12.5% overlap with Left Superior Temporal Gyrus					
25.2% overlap with Left Precentral gyrus (BA44)	1593	−44	13	7	3.12
20.5% overlap with Left Inferior Frontal Gyrus (p. Opercularis)					
14.4% overlap with Left Temporal Pole					
14.0% overlap with Left Inferior Frontal Gyrus (p. Triangularis)					
49.3% overlap with Right Rolandic Operculum (BA42)	1080	59	−14	12	3.12
42.3% overlap with Right Superior Temporal Gyrus					
60.1% overlap with Right Inferior Parietal Lobule (BA40)	702	62	−31	25	3.09
24.6% overlap with Right Superior Temporal Gyrus					
58.2% overlap with Right Superior Parietal Lobule (BA 7)	702	15	−73	56	3.22
37.4% overlap with Right Precuneus					

*The overlap is the percentage of voxels relative to the entire size of the cluster that overlapped with a given region's spatial map in the AFNI MNI atlas. So, for example, “40.9% overlap in Right Lingual Gyrus” means 40.9% of the cluster's entire volume was spatially located in the MNI region outlining the Right Lingual Gyrus*.

Similar to the correct trials, the brain activity associated with incorrect behavioral predictions of non-initially intended serves (compared to initially intended serves) revealed only one specific hemodynamic increase in the left cuneus extending to the left calcarine gyrus—a brain region associated with visual information processing. Different from the neuroimaging results for the correct trials, incorrect trials were also characterized by brain activity in right TPJ (right superior temporal gyrus) and left precentral gyrus/inferior frontal, and right IPL, but not in the right inferior frontal gyrus nor left IPL (Table 3; Figure 4). Indeed, compared to correct trials, incorrect trials were characterized by the absence of activations between IIS and NIIS in: (i) the caudate or thalamus, (ii) the right inferior frontal gyrus (an area known to be important in intention understanding; Iacoboni et al., 2005), (iii) left IPL, and (iv) EBA area.

#### fMRI correlational analyses

**Figure 5:**
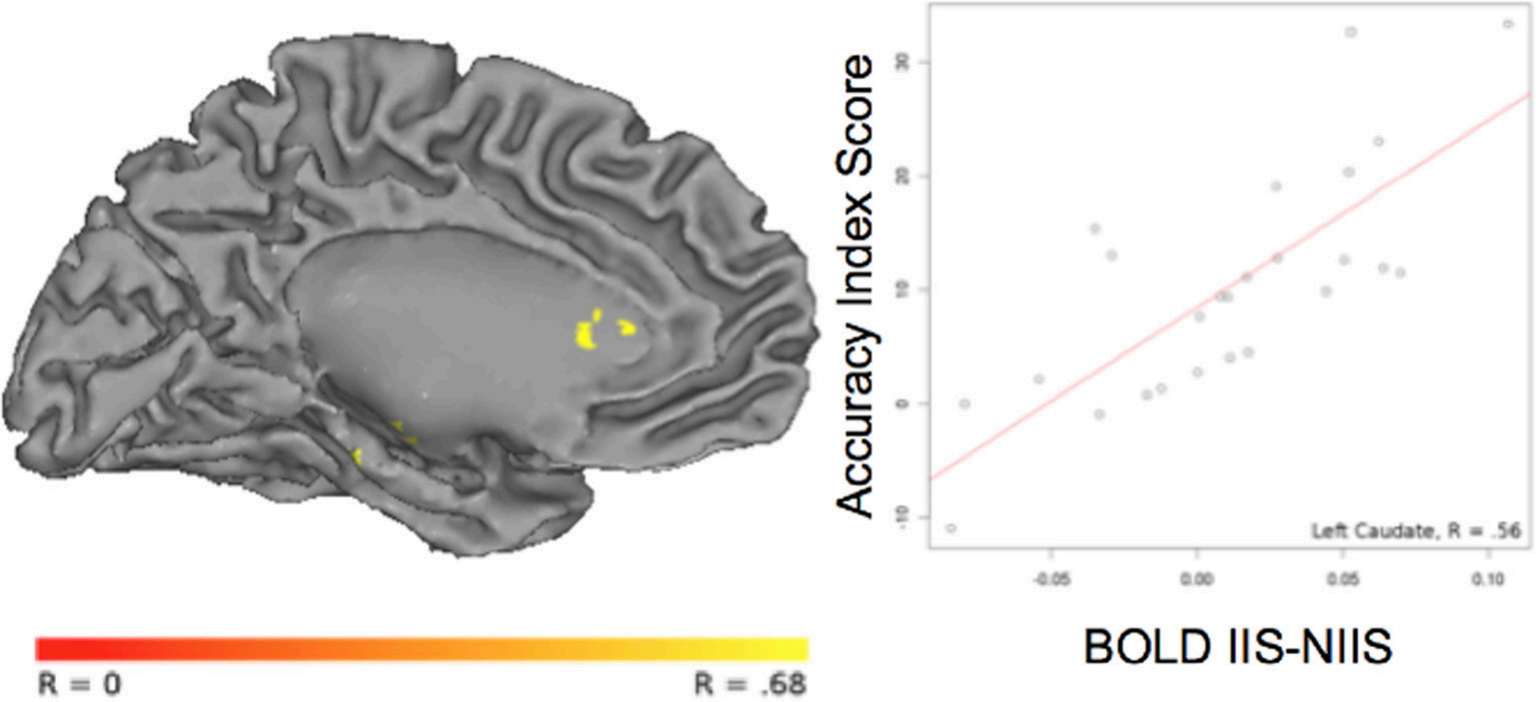
**Correlation between BOLD activity and behavioral accuracy index in the left caudate nucleus**. The correlation between the contrast of BOLD [IIS - NIIS] × accuracy index score (*r* = 0.56, *p* <.01, corrected) is projected onto the Caret AFNI Colin Brain surface model.

For *correct* trials, a positive correlation was observed between the accuracy index and BOLD IIS-NIIS contrast signal in dopaminergic-rich brain areas involved in procedural memory (such as the caudate nucleus; Figure 5), in the cerebellum and the left middle/superior temporal gyrus (Table 4), two areas known to be involved in analyses of motor movements and social cognition (Van Overwalle et al., 2014). Interestingly, correlations between *d*' accuracy index and BOLD IIS-NIIS contrast revealed a different pattern of regional brain activations (Table S2). This is in line with *d*' and simple accuracy measures reflecting different psychological mechanisms. More precisely, the accuracy index reflects correct trials whether by stimulus discriminability or response biases, whereas *d*' accuracy reflects stimulus discriminability *per se*. Although both indices were significant for the superior temporal gyrus, correlational results for the *d*' accuracy index selectively revealed greater activation in brain areas involved in attention, intention understanding (e.g., inferior frontal gyrus), perceptual discrimination (e.g., hypothalamus), and associative memory and perception of places and visual paths (e.g., parahippocampal region; Rajimehr et al., 2011; Table S2). Finally, no significant correlation was observed between the reaction times and BOLD IIS-NIIS contrast for correct trials (Table 4).

**Table 4:** **Correlations between BOLD [IIS – NIIS] for correct trials and behavioral measures**.

**BOLD [IIS – NIIS] for correct trials × Accuracy index**	**Vol (ul)**	***x***	***y***	***z***	***r***
15.1% overlap with Cerebellum	3105	0	−30	−21	0.57
8.8% overlap with Cerebellar Vermis					
8.4% overlap with Cerebellar Vermis					
57.7% overlap with Left Middle Temporal Gyrus (BA21)	2268	−47	−30	−2	0.57
9.5% overlap with Left Superior Temporal Gyrus					
30.5% overlap with Left Caudate Nucleus	1944	−8	17	16	0.56
19.9% overlap with Right Caudate Nucleus	918	19	−19	27	0.56
**BOLD [IIS – NIIS] for correct trials × Reaction times**	**Vol (ul)**	***x***	***y***	***z***	***r***
	NO CLUSTERS FOUND				

*The overlap is the percentage of voxels relative to the entire size of the cluster that overlapped with a given region's spatial map in the AFNI MNI atlas. So, for example, “15.1% overlap in Cerebellum” means 15.1% of the cluster's entire volume was spatially located in the MNI region outlining the Cerebellum*.

For *incorrect* trials, no significant correlations were observed between the accuracy index and BOLD IIS-NIIS contrast (Table 5), whereas a negative correlation was found in the left angular gyrus for the *d*' accuracy index (Table S3). This result suggests that the more participants made incorrect discriminations between the two conditions, the less this part of the brain was activated. The present results suggest that a reduced activity of this brain area, which is known to sustain various functions, such as self-other expansion, embodied cognition, and mental representation of past self-experiences, may play an important role in the commission of errors in tennis serve predictions. Finally, significant correlations were found for reaction times (Table 5). These correlations were a positive correlation in the right superior frontal gyrus and a negative correlation in the right parahippocampal gyrus (Table 5).

**Table 5:** **Correlations between BOLD [IIS – NIIS] for incorrect trials and behavioral measures**.

**BOLD [IIS – NIIS] for incorrect trials × Accuracy index**	**Vol (ul)**	***x***	***y***	***z***	***r***
	NO CLUSTERS FOUND				
**BOLD [IIS – NIIS] incorrect trials × Reaction times**	**Vol (ul)**	***x***	***y***	***z***	***r***
52.2% overlap with Right Superior Frontal Gyrus (BA8)	1809	4	34	44	0.56
51.0% overlap with Right Parahippocampal Gyrus (BA 30)	1026	30	−58	6	−0.57

*The overlap is the percentage of voxels relative to the entire size of the cluster that overlapped with a given region's spatial map in the AFNI MNI atlas. So, for example, “52.2% overlap in Right Superior Frontal Gyrus” means 52.2% of the cluster's entire volume was spatially located in the MNI region outlining the Right Superior Frontal Gyrus*.

***USTA levels***. A positive correlation was observed between USTA levels and BOLD activity for correct trials in the calcarine gyrus, extending to the lingual gyrus, left post-central gyrus, left thalamus, left caudate nucleus, right superior temporal gyrus, bilateral hippocampus, right para-hippocampal region, bilateral precuneus, left IFG, bilateral IPL, right angular gyrus, anterior cingulate and SMA (Table 6). Although no correlation was found between behavioral results and USTA levels, these findings are interesting as they suggest that the higher the USTA level of the participants was, the more intense was the activity in these brain regions involved in goal-directed motor actions, embodied cognition, attention, intention understanding, self-other expansion, and associative and procedural memory. No significant clusters were found between the USTA levels of the participants and their brain activity recruited during the difference scores of *incorrect* trials between IIS and NIIS (Table 7).

**Table 6:** **Correlations between tennis-related, self-reported measures, and BOLD [IIS – NIIS] contrast for correct trials**.

**BOLD [IIS – NIIS] x USTA level**	**Vol (ul)**	***x***	***y***	***z***	***r***
10.4% overlap with Left Calcarine Gyrus	47,466	−4	−46	−1	0.58
8.0% overlap with Left Lingual Gyrus					
6.4% overlap with Right Lingual Gyrus					
5.2% overlap with Left Calcarine Gyrus					
3.9% overlap with Left Fusiform Gyrus					
3.9% overlap with Left Middle Occipital Gyrus					
3.8% overlap with Left Hippocampus					
21.5% overlap with Left Postcentral Gyrus	9558	−25	−17	28	0.56
9.4% overlap with Left SupraMarginal Gyrus,					
5.6% overlap with Left Thalamus,					
5.4% overlap with Left Caudate Nucleus					
4.6% overlap with Left Middle Frontal Gyrus					
3.3% overlap with Left Inferior Parietal Lobule					
34.9% overlap with Right Superior Temporal Gyrus	6264	37	−22	−3	0.56
17.9% overlap with Right Hippocampus					
10.6% overlap with Right ParaHippocampal Gyrus					
6.8% overlap with Right Middle Temporal Gyrus					
5.4% overlap with Right Fusiform Gyrus					
3.3% overlap with Right Insula Lobe					
17.1% overlap with Right Parietal Lobe/Precuneus (BA 7)	3240	27	−65	29	0.56
16.3% overlap with Right Angular Gyrus					
14.7% overlap with Right Superior Occipital Gyrus					
14.6% overlap with Right Precuneus					
13.7% overlap with Right Cuneus					
3.8% overlap with Right Middle Temporal Gyrus					
3.4% overlap with Right Superior Parietal Lobule					
44.2% overlap with Right Postcentral Gyrus (BA3)	2619	43	−20	41	0.55
34.6% overlap with Right Precentral Gyrus					
3.5% overlap with Right Inferior Parietal Lobule					
33.8% overlap with Right Middle Cingulate Gyrus (BA 24)	1917	6	0	46	0.54
31.9% overlap with Right SMA					
13.7% overlap with Right SMA					
9.0% overlap with Middle Cingulate Cortex					
4.4% overlap with Right Superior Frontal Gyrus					
83.9% overlap with Left Parietal Lobule/Precuneus (BA 7)	1566	−22	−61	52	0.54
11.0% overlap with Left Inferior Parietal Lobule					
4.1% overlap with Left Precuneus					
24.2% overlap with Left Anterior Cingulate Cortex (BA32)	999	−23	33	12	0.57
3.6% overlap with Left Inferior Frontal Gyrus					
80.8% overlap with Right Precentral Gyrus (BA4)	918	58	−9	22	0.55
15.0% overlap with Right Rolandic Operculum					
**BOLD [IIS – NIIS] × Hours playing tennis per week**	**Vol (ul)**	***x***	***y***	***z***	***r***
65.2% overlap with Left Insula Lobe (BA 13)	729	−35	21	12	−0.57
33.5% overlap with Left Inferior Frontal Gyrus					
**BOLD [IIS – NIIS] × Hours watching tennis per week**	**Vol (ul)**	***x***	***y***	***z***	***r***
	NO CLUSTERS FOUND				
21.6% overlap with Right Pallidum	2916	15	−1	−3	–0.59
14.5% overlap with Right Caudate Nucleus					
5.8% overlap with Right Thalamus					
4.6% overlap with Right Hippocampus					
4.0% overlap with Right Amygdala					
51.8% overlap with Right Medial Frontal Gyrus (BA 9)	2268	4	41	19	0.60
38.7% overlap with Anterior Cingulate Cortex					
32.7% overlap with Right Insula (BA 13)	1458	38	8	−11	−0.60
12.0% overlap with Right Temporal Pole					
6.5% overlap with Right Middle Temporal Gyrus					
5.3% overlap with Right Superior Temporal Gyrus					
52.4% overlap with Left Insula (BA 13)	1404	−41	3	−11	−0.62
29.0% overlap with Left Superior Temporal Gyrus					
4.1% overlap with Left Middle Temporal Gyrus					
94.9% overlap with Left Middle Temporal Gyrus (BA 39)	1350	−53	−56	9	0.61
5.1% overlap with Left Superior Temporal Gyrus					
50.9% overlap with Right Anterior Prefrontal Cortex (BA10)	1134	28	49	21	0.59
39.7% overlap with Right Middle Frontal Gyrus					
51.2% overlap with Right Cerebellum	891	16	−52	−43	0.65
99.7% overlap with Right Cerebellum	891	32	−61	−30	0.58
68.0% overlap with Right Superior Temporal Gyrus (BA 22)	891	50	−18	8	0.60
28.5% overlap with Right Heschls Gyrus					
3.0% overlap with Right Rolandic Operculum					
100% overlap with Right Inferior Frontal Gyrus (BA 9)	891	52	15	21	0.56
96.7% overlap with Right Inferior Frontal Gyrus (BA 13)	783	43	29	4	0.56
67.0% overlap with Left Angular Gyrus (BA 39)	729	−38	−57	33	0.56
32.3% overlap with Left Inferior Parietal Lobule					

*The overlap is the percentage of voxels relative to the entire size of the cluster that overlapped with a given region's spatial map in the AFNI MNI atlas. So, for example, “67.0% overlap in Left Angular Gyrus” means 67.0% of the cluster's entire volume was spatially located in the MNI region outlining the Left Angular Gyrus*.

**Table 7:** **Correlations between tennis-related, self-reported measures, and BOLD [IIS – NIIS] contrast for incorrect trials**.

**BOLD [IIS – NIIS] × USTA level**	**Vol (ul)**	***x***	***y***	***z***	***r***
	NO CLUSTERS FOUND				
**BOLD [IIS – NIIS] × Hours playing tennis per week**	**Vol (ul)**	***x***	***y***	***z***	***r***
41.0% overlap with Right Superior Temporal Gyrus (BA41)	1161	42	−38	9	−0.59
**BOLD [IIS – NIIS] × Hours Watching Tennis per Week**	**Vol (ul)**	***x***	***y***	***z***	***r***
49.6% overlap with Right Angular Gyrus (BA 39)	5184	41	−60	22	−0.57
19.7% overlap with Right Middle Temporal Gyrus					
7.1% overlap with Right Middle Occipital Gyrus					
38.0% overlap with Left Cerebellum	3672	−25	−60	−33	−0.55
22.8% overlap with Left Cerebellum					
12.7% overlap with Left Fusiform Gyrus					
64.1% overlap with Left Precuneus (BA 7)	2889	−6	−69	41	−0.56
20.9% overlap with Left Superior Parietal Lobule					
13.0% overlap with Right Precuneus					
91.7% overlap with Right Middle Frontal Gyrus (BA 9)	2349	32	35	37	−0.59
7.2% overlap with Right Superior Frontal Gyrus					
30.5% overlap with Left Posterior Cingulate Cortex (BA 29)	2052	−3	−49	6	−0.63
16.7% overlap with Cerebellar Vermis					
10.7% overlap with Left Calcarine Gyrus					
82.0% overlap with Right Superior Parietal Lobule (BA 7)	1890	33	−55	57	−0.58
9.3% overlap with Right Postcentral Gyrus					
28.0% overlap with Left Middle Cingulate Gyrus (BA 23)	1647	0	−27	30	−0.55
7.7% overlap with Right Middle Cingulate Gyrus					
42.6% overlap with Right Caudate Nucleus	999	16	−10	24	−0.59
13.0% overlap with Right Thalamus					
61.4% overlap with Right Cerebellum	945	22	−54	−21	−0.55
23.9% overlap with Right Fusiform Gyrus					
14.5% overlap with Right Cerebellum					
32.4% overlap with Left Parahippocampal Area (BA 34)	945	−24	1	−9	−0.6
11.3% overlap with Left Putamen					
3.8% overlap with Right Postcentral Gyrus (BA 2)	864	31	−23	37	−0.55
1.5% overlap with Right SupraMarginal Gyrus					
88.4% overlap with Left Superior Temporal Gyrus (BA 13)	810	−54	−40	19	−0.6
11.6% overlap with Left SupraMarginal Gyrus					
**BOLD [IIS – NIIS] × Age first learned tennis**	**Vol (ul)**	***x***	***y***	***z***	***r***
21.4% overlap with Right Insula	1809	22	6	−10	−0.63
13.7% overlap with Right Amygdala					
5.4% overlap with Right Putamen					
54.1% overlap with Right Angular Gyrus (BA 39)	1809	42	−70	29	0.56
31.0% overlap with Right Middle Occipital Gyrus					
7.2% overlap with Right Middle Temporal Gyrus					
67.8% overlap with Right Cerebellum	1107	17	−52	−46	0.63
47.5% overlap with Right Middle Temporal Gyrus (BA 21)	1026	45	−2	−15	0.62
17.0% overlap with Right Superior Temporal Gyrus					
51.4% overlap with Right Hippocampus	1026	24	−16	−15	0.57
37.5% overlap with Right ParaHippocampal Gyrus					
92.9% overlap with Right Superior Temporal Gyrus (BA 42)	864	57	−29	13	−0.63
5.8% overlap with Right SupraMarginal Gyrus					
47.2% overlap with Left Superior Temporal Gyrus (BA 38)	756	−39	6	−12	−0.58
57.7% overlap with Right SupraMarginal Gyrus	729	54	−42	20	0.68
39.9% overlap with Right Superior Temporal Gyrus					

*The overlap is the percentage of voxels relative to the entire size of the cluster that overlapped with a given region's spatial map in the AFNI MNI atlas. So, for example, “47.2% overlap in Left Superior Temporal Gyrus” means 47.2% of the cluster's entire volume was spatially located in the MNI region outlining the Left Superior Temporal Gyrus*.

***Number of hours playing tennis per week***. A negative correlation was observed between this measure and the activity in a brain area involved in risk taking and decision making and simulation (insula; Paulus et al., 2003) for the difference scores of *correct* trials between IIS and NIIS (Table 6). These findings are preliminary but may suggest that the more participants reported playing tennis per week, the less the activity in these two brain areas when making correct tennis serve predictions—an interpretation that is consistent with the behavioral correlations we reported above.

For the difference scores of *incorrect* trials between IIS and NIIS, a negative correlation (*r* = −0.59, *p* <.05) was observed in the right superior temporal gyrus (BA 41; Table 7), suggesting that the more participants reported playing tennis per week, the less the activity in this particular brain areas when making incorrect tennis serve predictions.

***Number of hours watching tennis on TV per week***. No significant clusters were found between the number of hours participants reported watching tennis on TV and their brain activity recruited during the difference scores of *correct* trials between IIS and NIIS (Table 6). For *incorrect* predictions, however, a significant negative correlation was observed between this measure and the activity in their right angular gyrus (Table 7), which suggests that the more participants reported watching tennis on TV, the less their right angular gyrus was activated during their incorrect predictions. These results are consistent our above results suggesting a potential important role of the de-activation (or reduced activity) of the angular gyrus in incorrect tennis serve predictions. Finally, other negative correlations between the reported number of hours watching tennis on TV per week and activity in brain areas involved in motion, intention understanding, attention, and goal-directed actions (See Table 7 for details).

***Age of the participants when they first begun playing tennis***. For correct trials, a negative correlation was observed between this tennis-related measure and brain activity in the right pallidum, right caudate nucleus, and bilateral insula, whereas a positive correlation was observed between this measure and the activity in the right medial frontal gyrus, and brain areas involved in self-other representation, simulation and embodied cognition (see Table 6 for details). For incorrect trials, negative correlations were observed in the right insula, and bilateral superior temporal gyrus, although positive correlations were observed in the right angular gyrus, the right hippocampus, right supramarginal gyrus, and the right cerebellum (Table 7).

## Discussion

Anticipating intentions of an opponent during a fast interaction is a challenging problem. Our results reinforce and expand prior research by demonstrating that tennis experts are better at predicting where an expert server intends to serve (T or wide) when that expert server knows where he intends to serve *before* than *after* he tosses the ball in the air. Because the same action (a serve) can reflect different intentions (e.g., to serve to the T or the wide side of the service box) in tennis, the present study highlights the power of cognitive thinking *prior* action in interpersonal intention understanding. Although the participants were not able to explicitly articulate the reason of their successful tennis serve predictions, they were better at predicting IIS than NIIS.

These findings support predictions by the simulation and embodied cognition theories by demonstrating that the observers are more efficient in predicting one's intentions when that someone is pre-cognizant of their intentions before initiating their action (IIS condition) than when they don't know in advance their action intention (NIIS condition). In other words, when the observers share a common mental representation of action with the server, observers can more accurately read the intentions of the server. As demonstrated in previous research, this facilitation effect in reading another's intentions is positively correlated with active practice (as measured with the number of hours playing tennis per week) rather than passive practice (as measured with the number of hours watching tennis per week). Further studies could be done to specifically test the effects of the different components of a tennis profile and habits of a player on their anticipatory behaviors and performance. For instance, based on simulation and embodied cognition theories, one may be interested in comparing the effect of active tennis practice (e.g., playing tennis every week) vs. passive tennis practice (e.g., observing tennis on TV every week) on the accuracy and speed of serve predictions.

Our neuroimaging results extend these behavioral findings by demonstrating that accurate predictions were characterized by activation within both the Action Observation Network (AON including the hMNS) and the social brain network (SN). More precisely, our fMRI analyses of the IIS—NISS contrast for *correct* trials revealed activation of brain areas known to be involved in a broad variety of functions, including: (a) action prediction tasks (EBA in basketball athletes; Abreu et al., 2012), perception of body parts (Downing et al., 2001), limb movements, motor imagery and performance of motor action (Astafiev et al., 2004); (b) basic analysis of visual information and biological motion (calcarine gyrus, lingual gyrus; Servos et al., 2002); (c) attention and target discrimination (e.g., SPL; Capotosto et al., 2013); (d) retrieval of sensorimotor information and episodic memory (e.g., inferior parietal lobule; Sestieri et al., 2013); (e) action perspective from an egocentric viewpoint and intention understanding; (f) embodied cognition and simulation (e.g., inferior frontal gyrus, inferior parietal lobule; Grafton, 2009; Ortigue et al., 2009a, 2010a; Juan et al., 2013; Mazzarella et al., 2013); and (g) analysis of biological motion, agency, body parts, and perspective taking (STS and inferior parietal lobule; Buchel et al., 1998; Grezes et al., 2001; Ruby and Decety, 2001, 2003, 2004; Grossman and Blake, 2002; Ruby et al., 2002; Astafiev et al., 2004; Wright and Jackson, 2007). By demonstrating such a specific involvement of the inferior fronto-parietal network for *correct* trials (no similar constellation of brain activation was observed for *incorrect* trials; see Table 2), our present results reinforce Wright and Jackson (2007)'s results by highlighting the role of hMNS in tennis action prediction, and also reinforce simulation and embodied cognition theories (Grafton, 2009; Ortigue et al., 2009a, 2010a; Juan et al., 2013; Mazzarella et al., 2013), which suggest that reactivation of brain areas involved in own's motor performance and integration of past self experiences can facilitate fast and automatic visuo-motor matching process between what the observer sees and what they have executed (over and over) in the past.

Furthermore, the specific pattern of activation for *correct* IIS (compared to NIIS) suggests that accurate identification of a server's ultimate intentions regarding the direction of their serves operates through top-down processes as it builds on brain areas that have been previously recruited during observation and *performance* (practice) of that serve, adding regions associated with habit formation, body feature detection and performance. This interpretation also fits with the positive correlations found between some of these brain areas and the USTA levels. The closer the USTA level of the participants was to that of the model in the video, the more intense the activity was in these brain regions. To further investigate these correlations between USTA levels and top-down processes from these brain areas, future studies could be done with models having very high (expert) vs. very low (non-expert) USTA levels. Including these models with different USTA levels would allow to dissociate between motor and visual expertise.

In addition, IIS (compared to NIIS) was characterized by increased activity in dopaminergic-rich regions (bilateral thalamus, right putamen, right insula, and right caudate nucleus) involved in somatosensory integration, motivation, goal-directed actions as well as formation habits and procedural memory (Ashby and Crossley, 2010, 2012). The specific recruitment of a striatal based procedural memory in the understanding of intended serves is of particular interest as it reinforces previous studies highlighting the importance of procedural memory in embodied cognition and intention understanding (e.g., Altmann and Trafton, 2002; Grafton, 2009). Because the dorsal parts of the striatum, such as the caudate and putamen, are innervated by dopamine coming from both the ventral tegmental area and substantia nigra and going out to the insula also track rewarding stimuli of conditioned incentive value, the present results suggest that the recruitment of the dorsal striatum may be critical for the convergence between sensorimotor integration experience during both the practice of a tennis serve and the rewarding experience of predicting correctly an opponent's serve during prior matches. Further studies need to be done to test this hypothesis.

Finally, incorrect trials were associated with a different configuration of brain activation, which may provide clues as to when intention prediction goes wrong. Although overlapping areas of activations were observed within the brain areas involved in basic visual processing and spatial attention, no activation was observed during incorrect trials in brain areas involved in action prediction, embodied cognition, and procedural memory. Our study instead reveals that inaccurate predictions are related to activation in cortical areas known to be involved in low-level (bottom-up) computational processes associated with the sense of agency and self-other distinction as well as high-level processes such as theory of mind (Decety and Lamm, 2007). Notably, a specific activation was observed for incorrect trials in the right TPJ—a heteromodal association cortical area, emcompassing the supramarginal gyrus, posterior superior temporal gyrus, and the dorsal part of the occipital gyrus, which is involved both in low-level computational processes associated with theory of mind, perspective taking, a sense of agency, and higher social cognitive tasks (Decety and Lamm, 2007) and self-other distinction (Decety and Sommerville, 2003; Decety and Lamm, 2007). Further studies using high temporal resolution imaging methods (such as high density electrical neuroimaging) may help delineate the specific spatio-temporal dynamics of this brain area during inaccurate predictions of others' intentions. However, together the results of our study suggest experienced tennis players may make more accurate predictions of the service intentions of a skilled opponent when they focus on the somatic representation they observe rather than mentalizing about the strategy and explicit service intentions of their opponent.

### Conflict of interest statement

The authors declare that the research was conducted in the absence of any commercial or financial relationships that could be construed as a potential conflict of interest.

## Supplementary material

Movie 1 **IIS stimulus sample**. Example of a video clip in which the tennis player was told the intended location of his serve before he began his serve preparation.

Movie 2 **NIIS stimulus sample**. Example of a video clip in which the tennis player did not know in advance the location of his serve. Rather the experimenter told him where to serve when the ball was in the air.
