# Supplementary material for: Intention understanding over T: a neuroimaging study on shared representations and tennis return predictions
Source: Front Hum Neurosci. 2014 Oct 6;8:781. doi: 10.3389/fnhum.2014.00781 (PMC4186286; doi:10.3389/fnhum.2014.00781)
Supplement: Supplementary file 3 [file TableS1.DOC]

**Table S1. Correlation between Accuracy Index and tennis-related measures and reaction times.**

**Accuracy Index d' Accuracy Index**

**RT_all_** 0.17 0.20

**RT_IIS_** 0.15 0.21

**RT_NIIS_** 0.35 0.19

**USTA levels** 0.30 -0.02

**Hrs Playing Tennis / Week** 0.04 0*.40 **

**Hrs Watching Tennis / Week** -0.05 -0.02

**Age First Learned Tennis** 0.25 -0.34
