# Supplementary material for: Intention understanding over T: a neuroimaging study on shared representations and tennis return predictions
Source: Front Hum Neurosci. 2014 Oct 6;8:781. doi: 10.3389/fnhum.2014.00781 (PMC4186286; doi:10.3389/fnhum.2014.00781)
Supplement: Supplementary file 4 [file TableS2.DOCX]

Table S2. Correlations between BOLD [IIS – NIIS] for correct trials and d’ Accuracy Index

| **BOLD [IIS –NIIS] for correct trials x d' Accuracy Index** | **vol (ul)** | **x** | **y** | **z** | **R** |
| --- | --- | --- | --- | --- | --- |
| 11.5 % overlap with Right Hypothalamus | 5886 | 8 | -2 | -5 | 0.5968 |
| 4.4 % overlap with Left Olfactory cortex |  |  |  |  |  |
| 4.2 % overlap with Right Amygdala |  |  |  |  |  |
| 4.1 % overlap with Right ParaHippocampal Gyrus |  |  |  |  |  |
|  |  |  |  |  |  |
| 7.8 % overlap with Right Parahippocampal Gyrus (BA 30) | 3213 | 32 | -51 | 7 | 0.6109 |
| 4.3 % overlap with Right Middle Temporal Gyrus |  |  |  |  |  |
|  |  |  |  |  |  |
| 29.7 % overlap with Right Medial Frontal Gyrus (BA 9) | 2592 | 17 | 44 | 21 | 0.5875 |
| 26.8 % overlap with Right Superior Frontal Gyrus |  |  |  |  |  |
| 17.1 % overlap with Right Middle Frontal Gyrus |  |  |  |  |  |
| 13.5 % overlap with Left Anterior Cingulate Cortex |  |  |  |  |  |
|  |  |  |  |  |  |
| 74.7 % overlap with Left Superior Temporal Gyrus (BA 38) | 2376 | -39 | 8 | -16 | 0.6149 |
| 5.8 % overlap with Left Medial Temporal Pole |  |  |  |  |  |
| 5.2 % overlap with Left Superior Temporal Gyrus |  |  |  |  |  |
| 3.3 % overlap with Left Middle Temporal Gyrus |  |  |  |  |  |
|  |  |  |  |  |  |
| 47.8 % overlap with Right Precentral Gyrus (BA 9) | 1188 | 36 | 24 | 35 | 0.5775 |
| 45.5 % overlap with Right Middle Frontal Gyrus |  |  |  |  |  |
|  |  |  |  |  |  |
| 37.7 % overlap with Right Insula (BA 13) | 1161 | 33 | 14 | -9 | 0.5883 |
| 21.2 % overlap with Right Temporal Pole |  |  |  |  |  |
| 4.6 % overlap with Right Inferior Frontal Gyrus (p. Orbitalis) |  |  |  |  |  |
|  |  |  |  |  |  |
| 39.9 % overlap with Left Anterior Cingulate Cortex (BA 32) | 783 | -15 | 29 | 19 | 0.6442 |

Note: The overlap is the percentage of voxels relative to the entire size of the cluster that overlapped with a given region’s spatial map in the AFNI MNI atlas.  So, for example, “37.7% overlap in the right insula” means 37.7% of the cluster’s entire volume was spatially located in the MNI region outlining the right insula.
