# Supplementary material for: Intention understanding over T: a neuroimaging study on shared representations and tennis return predictions
Source: Front Hum Neurosci. 2014 Oct 6;8:781. doi: 10.3389/fnhum.2014.00781 (PMC4186286; doi:10.3389/fnhum.2014.00781)
Supplement: Supplementary file 5 [file TableS3.DOCX]

Table S3. Correlations between BOLD [IIS – NIIS] for incorrect trials and d’ Accuracy Index

**Incorrect Items - BOLD x d' Marshall Accuracy Index vol (ul) x y z R**

79.9 % overlap with Left Angular Gyrus (BA 39) 702 -35 -66 33 -0.58

14.9 % overlap with Left Middle Occipital Gyrus

5.2 % overlap with Left Inferior Parietal Lobule

Note: The overlap is the percentage of voxels relative to the entire size of the cluster that overlapped with a given region’s spatial map in the AFNI MNI atlas.  So, for example, “79.9% overlap in the right left angular gyrus” means 79.9% of the cluster’s entire volume was spatially located in the MNI region outlining the left angular gyrus.
